# Blood transfusion and the anaesthetist: management of massive haemorrhage

**DOI:** 10.1111/j.1365-2044.2010.06538.x

**Published:** 2010-11

**Authors:** D Thomas, M Wee, P Clyburn, I Walker, K Brohi, P Collins, H Doughty, J Isaac, PF Mahoney, L Shewry, Association of Anaesthetists of Great Britain and Ireland

## Abstract

1. Hospitals must have a major haemorrhage protocol in place and this should include clinical, laboratory and logistic responses.
2. Immediate control of obvious bleeding is of paramount importance (pressure, tourniquet, haemostatic dressings).
3. The major haemorrhage protocol must be mobilised immediately when a massive haemorrhage situation is declared.
4. A fibrinogen < 1 g.l^−1^ or a prothrombin time (PT) and activated partial thromboplastin time (aPTT) of > 1.5 times normal represents established haemostatic failure and is predictive of microvascular bleeding. Early infusion of fresh frozen plasma (FFP; 15 ml.kg^−1^) should be used to prevent this occurring if a senior clinician anticipates a massive haemorrhage.
5. Established coagulopathy will require more than 15 ml.kg^−1^ of FFP to correct. The most effective way to achieve fibrinogen replacement rapidly is by giving fibrinogen concentrate or cryoprecipitate if fibrinogen is unavailable.
6. 1:1:1 red cell:FFP:platelet regimens, as used by the military, are reserved for the most severely traumatised patients.
7. A minimum target platelet count of 75 × 10^9^.l^−1^ is appropriate in this clinical situation.
8. Group-specific blood can be issued without performing an antibody screen because patients will have minimal circulating antibodies. O negative blood should only be used if blood is needed immediately.
9. In hospitals where the need to treat massive haemorrhage is frequent, the use of locally developed shock packs may be helpful.
10. Standard venous thromboprophylaxis should be commenced as soon as possible after haemostasis has been secured as patients develop a prothrombotic state following massive haemorrhage.

## Introduction

There are an increasing number of severely injured patients who present to hospital each year. Trauma is the leading cause of death in all ages from 1 to 44 years. Haemorrhagic shock accounts for 80% of deaths in the operating theatre and up to 50% of deaths in the first 24 h after injury. Only 16% of major emergency departments in the UK use a massive haemorrhage guideline [1].

The management of massive haemorrhage is usually only one component of the management of a critically unwell patient. These guidelines are intended to supplement current resuscitation guidelines and are specifically directed at improving management of massive haemorrhage [2]. The guidance is intended to provide a better understanding of the priorities in specific situations. Effective teamwork and communication are an essential part of this process.

Definitions of massive haemorrhage vary and have limited value. The Working Party suggests that the nature of the injury will usually alert the anaesthetist to the probability of massive haemorrhage and can be arbitrarily considered as a situation where 1–1.5 blood volumes may need to be infused either acutely or within a 24-h period.

The formulation of guidance in the style of previous AAGBI guidelines has been difficult in such a rapidly changing area. The grade of evidence has not been mentioned within the text, but the editing of the final draft and Working Party membership have been cross-checked with other recently published documents. The Working Party believes that at the current time, its advice is consistent with recently published European guidelines and the availability of current evidence [3–5].

It is envisaged that the website version of this document will be updated at least annually and earlier if an addendum or correction is deemed urgent.

## Organisational aspects

Hospitals must have a major haemorrhage protocol in place and this should include clinical, laboratory and logistic responses. Protocols should be adapted to specific clinical areas. It is essential to develop an effective method of triggering the appropriate major haemorrhage protocol.

### Roles within a team

#### Team leader

The team leader is the person who declares a massive haemorrhage situation; this is usually the consultant or the most senior doctor at the scene. Their role is to direct and co-ordinate the management of the patient with massive haemorrhage.

#### Communication

The team leader should appoint a member of the team as communications lead whose sole role is to communicate with the laboratories and other departments.

#### Collection of blood samples, blood and components

A member of the team should be allocated to convey blood samples, blood and blood components between the laboratory and the clinical area. This role is usually taken by a porter or healthcare support worker who should ideally be in constant radio communication with the team. In their absence, a nurse or doctor should be identified to take on this role.

#### Securing intravenous and central access

A member of the team should be identified whose role is to secure intravenous access, either peripherally or centrally. Large-bore 8-Fr. central access is the ideal in adults; in the event of failure, intra-osseous or surgical venous access may be required.

#### Switchboard

The switchboard must alert certain key clinical and support people when a massive haemorrhage situation is declared. These include

- Hospital Transfusion Laboratory Biomedical Scientist (BMS) or equivalent
- Coagulation Biomedical Scientist (BMS) or equivalent
- Haematologist on call
- ICU senior doctor on site
- ICU Nurse in charge
- Surgical senior doctor on site
- Radiologist on call

## Dealing with the patient with massive haemorrhage

There are both clinical and logistic issues to consider. These include clinical management of the patient, setting processes in place to deliver blood and blood components to the patient, and organisation of emergency interventions to stop the bleeding (surgical or radiological). There are two common scenarios:

### A massively bleeding/injured/ill patient en route

With warning, resources and personnel can be mobilised to be in position to receive the patient. A brief history can alert the team to the risk of massive bleeding:

- History of trauma (blunt or penetrating)
- Obstetric patient
- Major surgery (neurosurgery, spinal, cardiac, liver surgery)
- Underlying medical condition affecting coagulation

### Presentation of a patient with minimal or no notice

This is the common scenario in the accident and emergency department. Here, the immediate concern is hands-on management of the patient:

- Stop any external bleeding
- Assess the patient and treat
- Trigger massive haemorrhage protocol
- Move to the next appropriate level of care

### Immediate actions in dealing with a patient with massive haemorrhage

- Control obvious bleeding points (pressure, tourniquet, haemostatic dressings)
- Administer high *F*_I_o_2_
- iv access – largest bore possible including central access
- If patient is conscious and talking and a peripheral pulse is present, the blood pressure is adequate.
- Baseline bloods – full blood count (FBC), prothrombin time (PT), activated partial thomboplastin time (aPTT), Clauss fibrinogen* and cross-match.
- If available, undertake near-patient testing e.g. thromboelastography (TEG) or thromboelastometry (ROTEM).
- Fluid resuscitation – in the massive haemorrhage patient, this means warmed blood and blood components. In terms of time of availability, blood group O is the quickest, followed by group specific, then cross-matched blood.
- Actively warm the patient and all transfused fluids.
- Next steps: rapid access to imaging (ultrasound, radiography, CT), appropriate use of focused assessment with sonography for trauma scanning and/or early whole body CT if the patient is sufficiently stable, or surgery and further component therapy.
- Alert theatre team about the need for cell salvage autotransfusion.

*A derived fibrinogen is likely to be misleading and should not be used.

### Ongoing assessment

- Look at injury patterns
- Look for obvious blood loss (on clothes, on the floor, in drains)
- Look for indications of internal blood loss
- Assess physiology (skin colour, heart rate, blood pressure, capillary refill, conscious level)

Some patients compensate well despite significant blood loss. A rapid clinical assessment will give very strong indications of those at risk. It is important to restore organ perfusion, but it is not necessary to achieve a normal blood pressure at this stage [6–8].

### Further management

Once control of bleeding is achieved, aggressive attempts should be made to normalise blood pressure, acid-base status and temperature, but vasopressors should be avoided. Active warming is required. Coagulopathy should be anticipated and, if possible, prevented. If present, it should be treated aggressively (see Dealing with coagulation problems).

Surgery must be considered early. However, surgery may have to be interrupted and limited to ‘damage control’. Once bleeding has been controlled, abnormal physiology can be corrected [9–11].

Following treatment for massive haemorrhage, the patient should be admitted to a critical care area for monitoring and observation, and monitoring of coagulation, haemoglobin and blood gases, together with wound drain assessment to identify overt or covert bleeding.

### Venous thromboprophylaxis

Standard venous thromboprophylaxis should be commenced as soon as possible after bleeding has been controlled, as patients rapidly develop a prothrombotic state. Temporary inferior vena cava filtration may be necessary.

## Dealing with coagulation problems

### Haemostatic defects in massive haemorrhage

The haemostatic defect in massive haemorrhage will vary, depending on the amount and cause of bleeding and underlying patient-related factors. It is likely to evolve rapidly. Patient management should be guided by laboratory results and near-patient testing, but led by the clinical scenario.

#### Dilutional coagulopathy

All patients being treated for massive haemorrhage are at risk of dilutional coagulopathy leading to reduced platelets, fibrinogen and other coagulation factors. This occurs if volume replacement is with red cells, crystalloid and plasma expanders, and insufficient infusion of fresh frozen plasma (FFP) and platelets. Dilutional coagulopathy should be prevented by early infusion of FFP.

#### Consumptive coagulopathy

Some patients with massive haemorrhage are also at risk of a consumptive coagulopathy and are liable to develop haemostatic failure without significant dilution. Consumption is commonly seen in obstetric haemorrhage, particularly associated with placental abruption and amniotic fluid embolus, in patients on cardiopulmonary bypass (CPB), following massive trauma especially involving head injury, and in the context of sepsis.

*Activation of anticoagulant pathways* is associated with massive trauma and patients may have haemostatic compromise without abnormal coagulation tests [12].

*Platelet dysfunction* is associated with CPB, renal disease and anti-platelet medication.

*Hyperfibrinolysis* is particularly associated with obstetric haemorrhage, CPB and liver surgery.

### Anticoagulant drugs

In the context of massive haemorrhage, warfarin should be reversed with a prothrombin complex concentrate (PCC) and intravenous vitamin K (5–10 mg). The dose is dependent on the international normalised ratio (INR) (see Table 1).

**Table 1:** Possible regimen for prothrombin complex concentrate (PCC)

INR	Dose of PCC; u.kg^−1^
2–3.9	25
4–5.9	35
> 6	50

INR, international normalised ratio.

*Unfractionated heparin* can be reversed with protamine (1 mg protamine reverses 100 u heparin). Excess protamine induces a coagulopathy. Usual reversal is by infusing either 25 or 50 mg of intravenous protamine.

*Low molecular weight heparin* can be partially reversed with protamine.

*Direct thrombin and factor Xa inhibitors* e.g. fondaparinux, dabigatran and rivaroxaban cannot be reversed.

### Aspirin and P2Y12 antagonists

Patients taking aspirin have a low risk of increased bleeding, whilst those on P2Y12 antagonists have a higher risk. The anti-platelet effect of aspirin can be reversed by platelet transfusion, but the effect of the P2Y12 antagonist, clopidogrel, is only partially reversed by platelets.

### Inherited bleeding disorders

It is very likely that patients with an inherited bleeding disorder will be registered with a haemophilia centre and urgent advice should be sought if they present with massive haemorrhage.

### Liver disease

Liver disease is associated with decreased production of coagulation factors, natural anticoagulants and the production of dysfunctional fibrinogen (dysfibrinogenaemia). It should be anticipated that these patients will develop a clinically significant dilutional coagulopathy and haemostatic failure with bleeds less than one blood volume.

### Interpretation of laboratory tests

A fibrinogen < 1 g.l^−1^ or a PT and aPTT > 1.5 times normal represents an established haemostatic failure and is predictive of microvascular bleeding. Early infusion of FFP should be used to prevent this occurring if a senior clinician anticipates a massive haemorrhage.

*Clauss fibrinogen* is an easily available test and should be specifically requested if not part of the routine coagulation screen. The fibrinogen level is more sensitive than the PT and aPTT to a developing dilutional or consumptive coagulopathy. Levels below 1 g.l^−1^, in the context of massive haemorrhage, are usually insufficient, and emerging evidence suggests that a level above 1.5 g.l^−1^ is required. Higher levels are likely to improve haemostasis further.

*A platelet count* below 50 × 10^9^.l^−1^ is strongly associated with haemostatic compromise and microvascular bleeding in a patient being treated for massive haemorrhage. A minimum target platelet count of 75 × 10^9^.l^−1^ is appropriate in this clinical situation.

*The PT* is an insensitive test for haemostatic compromise and a relatively normal result should not necessarily reassure the clinician. It is common practice to correct to PT to within 1.5 of normal; however, this may not be an appropriate target in many situations [13].

*An INR* is not an appropriate test in massive haemorrhage because it is standardised for warfarin control, and results may be misleading in the context of dilutional and consumptive coagulopathies and liver disease.

*The aPTT* is commonly used to guide blood product replacement but, as with the PT, correcting to 1.5 times normal is not necessarily an appropriate strategy because haemostatic failure may already be significant at this level. The aPTT should be maintained below 1.5 times normal as the minimum target.

If whole blood point of care testing is used, a protocol for blood product usage based on thromboelastogram (TEG/ROTEM) results should be agreed in advance.

Haemostatic tests and FBC should be repeated at least every hour if bleeding is ongoing, so that trends may be observed and adequacy of replacement therapy documented. Widespread microvascular oozing is a clinical marker of haemostatic failure irrespective of blood tests and should be treated aggressively.

### Management of haemostasis

The coagulopathy during massive haemorrhage is likely to evolve rapidly and regular clinical review and blood tests are required. It is important to anticipate and prevent haemostatic failure, but if haemostatic failure has occurred, standard regimens (e.g. FFP 15 ml.kg^−1^) can be predicted to be inadequate and larger volumes of FFP are likely to be required.

### Prevention of coagulopathy

Emerging evidence supports the early use of FFP to prevent dilutional coagulopathy. If an experienced clinician anticipates a blood loss of one blood volume, FFP should be infused to prevent coagulopathy. While FFP 15 ml.kg^−1^ is appropriate for uncomplicated cases, increased volumes of FFP will be needed if a consumptive coagulopathy is likely or the patient has underlying liver disease.

A minimum target platelet count of 75 × 10^9^.l^−1^ is appropriate in this clinical situation.

1:1:1 red cell:FFP:platelet regimens, as used by the military, are reserved for the most severely traumatised patient and are not routinely recommended [14, 15].

### Treatment of haemostatic failure

In the context of massive haemorrhage, patients with widespread microvascular oozing or with coagulation tests that demonstrate inadequate haemostasis (fibrinogen < 1 g.l^−1^ or PT/aPTT > 1.5 above normal), should be given FFP in doses likely to correct the coagulation factor deficiencies. This will require more than 15 ml.kg^−1^, and at least 30 ml.kg^−1^ would be a reasonable first-line response [16, 17].

Platelets should be maintained at at least 75 × 10^9^.l^−1^ [2, 18].

Although it is often recommended that hypofibrinogenaemia unresponsive to FFP be treated with cryoprecipitate, treatment may be associated with delays because of thawing and transportation.

Fibrinogen replacement can be achieved much more rapidly and predictably with fibrinogen concentrate (no requirement for thawing as for cryoprecipitate) given at a dose of 30–60 mg.kg^−1^. This product is not currently licensed in the UK and must be given on a named patient basis. Fibrinogen concentrate is licensed in many European countries to treat both congenital and acquired hypofibrinogenaemia [19].

#### Hyperfibrinolysis

Intravenous tranexamic acid should be used in clinical situations where increased fibrinolysis can be anticipated. Support for its use has strengthened recently with the positive report of its use in traumatic haemorrhage [20] (see Other interventions on pharmacological management of massive haemorrhage).

Hypocalcaemia and hypomagnesaemia are often associated with massively transfused patients and will need monitoring and correction.

#### rFVIIa

This drug has been used for treatment of massive haemorrhage unresponsive to conventional therapy. Recent review of data has highlighted the risk of arterial thrombotic complications and the specification of product characteristics now states: ‘Safety and efficacy of NovoSeven (rFVIIa) have not been established outside the approved indications and therefore NovoSeven should not be used’. Where centres decide to use this therapy, local protocols must be agreed in advance. rFVIIa is usually given with tranexamic acid and is not as efficacious if the patient has a low fibrinogen.

Some centres use PCC (concentrated factors II, VII, IX and X) in certain clinical situations such as liver disease and post-CPB; local protocols must be agreed in advance.

## Logistics of blood supply

### Identification

Positive patient identification is essential at all stages of the blood transfusion process and a patient should have two identification bands in situ. The healthcare professional administering the blood component must perform the final administrative check for every component given. All persons involved in the administration of blood must be trained and certificated in accordance with national standards.

### Standard issue

Pre-transfusion procedures are designed to determine the patient’s ABO and Rhesus D (RhD) status, to detect red cell antibodies that could haemolyse transfused cells and confirm compatibility with each of the units of red cells to be transfused. Red cell selection may be based on a serological cross-match or electronic issue. Standard issue of red cells may take approximately 45 min.

### Emergency issue

*Group O RhD negative* is the blood group of choice for transfusion of red cells in an emergency where the clinical need is immediate. However, overdependence on group O RhD negative red cells may have an adverse impact on local and national blood stock management and it is considered acceptable to give O RhD positive red cells to male patients.

Hospitals should avoid the need for elective transfusion of group O RhD negative red cells to non-O RhD negative recipients. Clinical staff should endeavour to provide immediate blood samples for grouping in order to allow the use of group specific blood.

In the emergency situation, blood can be issued following identification of group without knowing the result of an antibody screen –‘group specific blood’. Grouping can be performed in about 10 min, not including transfer time, and group specific blood can be issued. This of course is a higher risk strategy and depends on the urgency for blood. In massive bleeding, patients will have minimal circulating antibodies, so will usually accept group specific blood without reaction. If the patient survives, antibodies may develop at a later stage.

Women who are RhD negative and of childbearing age, who are resuscitated with Rh D positive blood or platelets, can develop immune anti-D, which can cause haemolytic disease of the newborn in subsequent pregnancies. To prevent this, a combination of exchange transfusion and anti-D can be administered, on the advice of a haematologist, within 72 h of the transfusion.

### Blood storage and transfer

Cold chain requirements are essential under European Law. Blood should be transfused within 4 h of leaving a controlled environment. Blood issued cannot be returned to stock if out of a controlled temperature and monitored fridge for longer than 30 min. If blood is issued within a correctly packed and validated transport box, the blood should be placed back in a blood fridge normally within 2 h, providing the box is unopened. Blood transfusion laboratory staff will then assess the acceptability of blood for return to stock. Only in exceptional circumstances should blood components be transferred with a patient between hospital trusts or health boards.

### Traceability

It is a statutory requirement that the fate of all blood components must be accounted for. These records must be held for 30 years. Staff must be familiar with local protocols for recording blood use in clinical notes and for informing the hospital transfusion laboratory.

*The hospital transfusion committee* is the ideal forum to allow cross-specialty discussions about protocols and organisation for dealing with massive haemorrhage. Audit of previous instances allows a refinement of response to ensure efficient and timely treatment. This level of organisation can only be arranged at a local level.

*Stock management* of labile components when there is unpredictable demand is a challenge. Large stock-holding is associated with wastage, whereas insufficient stocks may lead to clinical disaster.

Most hospitals rely on rapid re-supply of platelets from the Blood Service rather than holding stocks. Anaesthetists need to be aware of local arrangements and the normal time interval for obtaining platelets in an emergency.

### Blood shortages

National demand for blood components may exceed supply. National blood shortage plans will be activated in the event of red cell and platelet shortages. Guidance is given for the prioritisation of patient groups during shortage. The transfusion support during massive haemorrhage is a priority. However, it is expected that all efforts be made to stop the bleeding and reduce the need for donor blood. The use of cell salvage is encouraged in all cases of massive haemorrhage (Further information is available in *Blood Transfusion and the Anaesthetist – Intra-operative Cell Salvage.* AAGBI: http://www.aagbi.org/publications/guidelines/docs/cell%20_salvage_2009_amended.pdf).

## Blood components

This section advises on the appropriate use of blood components during massive haemorrhage (see Dealing with coagulation problems). This advice is required because red cell concentrates do not contain coagulation factors or platelets.

Patients with massive haemorrhage may require all blood components. Blood may be required not just at the time of resuscitation, but also during initial and repeat surgery. The benefits of timely and appropriate transfusion support in this situation outweigh the potential risks of transfusion and may reduce total exposure to blood components.

(Further information is available in *Blood Transfusion and the Anaesthetist – Blood Component Therapy*. AAGBI: http://www.aagbi.org/publications/guidelines/docs/bloodtransfusion06.pdf).

### Paediatric components

A comprehensive guideline for neonatal and paediatric transfusion together with a recent update statement is available at http://www.bcshguidelines.com/. Useful principles are: minimise and stop blood loss; minimise donor exposure; and use paediatric components where readily available (See Table 2).

**Table 2:** Blood component volumes and rates of administration for infants and children

Component	Volume
Red cell concentrates	Vol (ml) = desired Hb rise (g.dl^−1^) × wt (kg) × 3
Platelets	Children < 15 kg (10–20 ml.kg^−1^)Children > 15 kg (1 adult bag)
FFP (MB treated)	10–20 ml.kg^−1^
Cryoprecipitate	5–10 ml.kg^−1^ (usually max 10 units – approx 300 ml)

Vol, volume; Hb, haemoglobin; FFP, fresh frozen plasma; MB, methylene blue.

## Equipment to aid transfusion

### Giving sets, filters and pressure infusion

All blood components should be administered using a blood component administration set, which incorporates a 170–200 μm filter.

There is no current need to use any sort of additional filter in massive haemorrhage when using allogeneic product, as pre-storage leucodepletion has rendered this process unnecessary. If red cell salvage is being used, a 40-μm filter may still be indicated e.g. if small bone fragments contaminate the surgical field.

Although a special platelet giving set is ideally used for platelet transfusion, it is unnecessary in massive haemorrhage. The important issue is to administer platelets via a clean 170–200 μm giving set (see below), as one that has previously been used for red cells may cause the platelets to stick to the red cells and therefore reduce the effective transfused platelet dose.

The use of an adequate warming device is recommended in massively bleeding patients and this equipment needs to be available in all emergency rooms and theatre suites, allowing adequate warming of administered blood at high infusion rates.

### Infusion devices

- Only blood component administration sets that are compatible with the infusion device should be used (check manufacturers’ recommendations). Infusion devices should be regularly maintained and any adverse outcome as a result of using an infusion device to transfuse red cells should be reported to the Medical Devices section of the Medicines and Healthcare products Regulatory Agency (MHRA).
- Administration sets used with infusion devices should incorporate an integral mesh filter (170–200 μm).
- The pre-administration checking procedure should include a check of the device and device settings.

### Infusion rate devices

- Either gravity or electronic infusion devices may be used for the administration of blood and blood components. Infusion devices allow a precise infusion rate to be specified.
- Rapid infusion devices may be used when large volumes have to be infused quickly, as in massive haemorrhage. These typically have a range of 6–30 l.h^−1^ and usually incorporate a blood-warming device.
- Infusion devices should only be used if the manufacturer verifies them as safe for this purpose and they are CE-marked.
- The volume delivered should be monitored regularly throughout the infusion to ensure that the expected volume is delivered at the required rate.

### Pressure devices

- External pressure devices make it possible to administer a unit of red cells within a few minutes. They should only be used in an emergency situation together with a large-gauge venous access cannula or device.
- External pressure devices should:
  - Exert pressure evenly over the entire bag;
  - Have a gauge to measure the pressure;
  - Not exceed 300 mmHg of pressure;
  - Be monitored at all times when in use.

### Blood warmers

- In all adults undergoing elective or emergency surgery (including surgery for trauma) under general or regional anaesthesia, ‘intravenous fluids (500 ml or more) and blood components should be warmed to 37 °C’ [21, 22]. The greatest benefit is from the controlled warming of red cells (stored at 4 °C) rather than platelets (stored at 22 ± 2 °C) or FFP/cryoprecipitate (thawed to 37 °C) [23]. Of note, there is no evidence to suggest that infusion of platelets or FFP through a blood warmer is harmful.
- In most other clinical situations where there is concern, it is sufficient to allow blood to rise to ambient temperature before transfusion. Special consideration should be given when rapidly transfusing large volumes to neonates, children, elderly patients and patients susceptible to cardiac dysfunction.
- Blood should only be warmed using approved, specifically designed and regularly maintained blood warming equipment with a visible thermometer and audible warning. Settings should be monitored regularly throughout the transfusion.
- Blood components should never be warmed using improvisations, such as putting the pack in warm water, in a microwave or on a radiator.

## Other interventions

### Pharmacological management options

#### Antifibrinolytics

Fibrinolysis is the process whereby established fibrin clot is broken down. This can occur in an accelerated fashion, destabilising effective coagulation in many clinical situations associated with massive haemorrhage, including multiple trauma, obstetric haemorrhage and major organ surgery (e.g. cardiothoracic, liver) including transplantation surgery.

Accelerated fibrinolysis can be identified by laboratory assay of d-dimers or fibrin degradation products, or by use of coagulation monitors such as TEG or ROTEM. It is accepted that not all hospitals can provide either the hardware or expertise to interpret TEG and ROTEM.

Antifibrinolytic drugs, such as tranexamic acid, have been used to reverse established fibrinolysis in the setting of massive blood transfusion.

Whilst systematic reviews fail to demonstrate evidence from randomised controlled trials to support the routine use of antifibrinolytic agents in managing massive haemorrhage, they are considered effective if accelerated fibrinolysis is identified.

*Tranexamic acid* inhibits plasminogen activation, and at high concentration inhibits plasmin. The recent CRASH-2 trial supports its use at a loading dose of 1 g over 10 min followed by 1 g over 8 h [20]. There are few adverse events or side effects associated with tranexamic acid use in the setting of massive haemorrhage [20]. Repeat doses should be used with caution in patients with renal impairment, as the drug is predominantly excreted unchanged by the kidneys. It is contraindicated in patients with subarachnoid haemorrhage, as anecdotal experience suggests that cerebral oedema and cerebral infarction may occur.

*Aprotinin* is a serine protease inhibitor, inhibiting trypsin, chymotrypsin, plasmin and kallikrein. It has been used to reduce blood loss associated with accelerated fibrinolysis in major surgery (e.g. cardiothoracic surgery, liver transplantation). Recently, there have been concerns about the safety of aprotinin. Anaphylaxis occurs at a rate of 1:200 in first-time use. A study performed in cardiac surgery patients reported in 2006 showed that there was indeed a risk of acute renal failure, myocardial infarction and heart failure, as well as stroke and encephalopathy [24]. As a result of this, and other follow-up work, the MHRA recommends that aprotinin should only be used when the likely benefits outweigh any risks to individual patients. As a result, use of aprotinin is now limited to highly specialised surgical situations, e.g. cardiac and liver transplantation, and is used on a named patient basis only.

#### Factor concentrates

Coagulation factor concentrates may be required for patients with inherited bleeding disorders such as haemophilia or von Willebrand disease. They should only be used under the guidance of a haemophilia centre. (For recombinant factor VIIa, prothrombin complex concentrate and fibrinogen concentrate, see Dealing with coagulation problems).

### Non-pharmacological management options

#### Radiologically aided arterial embolisation

These techniques are becoming more widespread and successful cessation of bleeding can be achieved with embolisation of bleeding arteries following angiographic imaging. The suitability of such manoeuvres needs to be assessed in each individual case and will also depend on availability of an interventional radiologist. The technique can be remarkably effective and may eliminate the need for surgical intervention, particularly in major obstetric haemorrhage.

#### Cell salvage

The use of intra-operative cell salvage can be very effective at both reducing demand on allogeneic supplies and providing a readily available red cell supply in massive haemorrhage. National Institute of Health and Clinical Excellence (NICE) guidelines have also supported its use where large blood loss is experienced in obstetric haemorrhage and complex urological surgery such as radical prostatectomy. The indications for cell salvage are detailed in *Blood Transfusion and the Anaesthetist – Intra-operative Cell Salvage* (http://www.aagbi.org/publications/guidelines/docs/cell%20_salvage_2009_amended.pdf).
